# Tangier Disease

**DOI:** 10.1007/s11883-026-01457-5

**Published:** 2026-09-19

**Authors:** Sabitha Sasidharan Pillai, Preneet Cheema Brar, Ambika P. Ashraf

**Affiliations:** 1 Center for Endocrinology, Diabetes and Metabolism, Children’s Hospital Los Angeles, Los Angeles, CA USA; 2 Department of Pediatrics, Keck School of Medicine, University of Southern California, Los Angeles, CA USA; 3 Division of Endocrinology and Diabetes, New York University Grossman Long Island School of Medicine, Mineola, NY USA; 4 Department of Pediatrics, The University of Alabama at Birmingham, Birmingham, AL USA

**Keywords:** Tangier disease, Hypoalphalipoproteinemia, Yellow orange tonsils, Peripheral neuropathy

## Abstract

**Purpose of Review:**

We describe two patients with Tangier disease (TD) and provide a narrative review of its clinical features, genetics, diagnosis, and management. A literature search of PubMed and Google Scholar was performed to identify English-language publications on TD, and relevant articles were reviewed narratively.

**Recent Findings:**

Patient # 1 was diagnosed with TD due to compound heterozygous variant in *ABCA1* (one pathogenic variant (c.1759 C > T, p.Arg587Trp) and one variant of uncertain significance (VUS) (c.1733 C > G, p.Pro578Arg)) when evaluated for histopathological findings of tonsillectomy specimen suggestive of TD. Patient # 2 was found to have TD due to homozygous pathogenic variant in *ABCA1* (c.1769G > T p. Trp590Leu) when evaluated for incidental detection of low high density lipoprotein (HDL-C).

**Summary:**

TD, characterized by HDL-C deficiency affects reverse cholesterol transport causing intracellular cholesterol accumulation in various tissues producing characteristic clinical presentation. There is no definite treatment for TD. To the best of our knowledge the VUS noted in patient # 1 is likely pathogenic. This report will aid a variant reclassification.

## Introduction

Tangier disease (TD) is an autosomal recessive monogenic disorder with an approximate prevalence of 1 in 640,000 globally based on allele frequencies of responsible gene, *ABCA1(adenosine triphosphate binding cassette subfamily A member 1)* [1]. TD is distinguished by marked deficiency or absence of high-density lipoprotein cholesterol (HDL-C) (hypoalphalipoproteinemia) and apolipoprotein A-1 (ApoA-I) [1]. TD was initially described by Fredrickson et al. in 1964 when he described two children from Tangier Island in Chesapeake Bay, Virginia, United States who presented with enlarged orange tonsils, hepatosplenomegaly, corneal opacities and very low HDL-C [2]. Later in 1999 it was identified that homozygous or compound heterozygous pathogenic variant in *ABCA1* on chromosome 9q31 caused TD [3]. The long-term consequences of TD are variable and heterogeneous, driven by progressive cholesterol ester accumulation in macrophages and the reticuloendothelial system due to dysfunctional ABCA1-mediated cholesterol efflux [1]. Given the rarity of TD and the wide heterogeneity in clinical presentation, particularly in pediatric patients, we present two contrasting cases to highlight diagnostic challenges, phenotypic variability, and the importance of integrating clinical, biochemical, histopathologic, and genetic data in establishing the diagnosis. A literature search of PubMed and Google Scholar was performed to identify publications on TD published through 2025. Articles were selected based on their relevance to the clinical features, biochemical findings, genetic basis, and management of TD and were reviewed narratively. Non-English-language publications and articles not directly relevant to TD were excluded.

## Patients with TD

### Patient 1

**Fig. 1:**
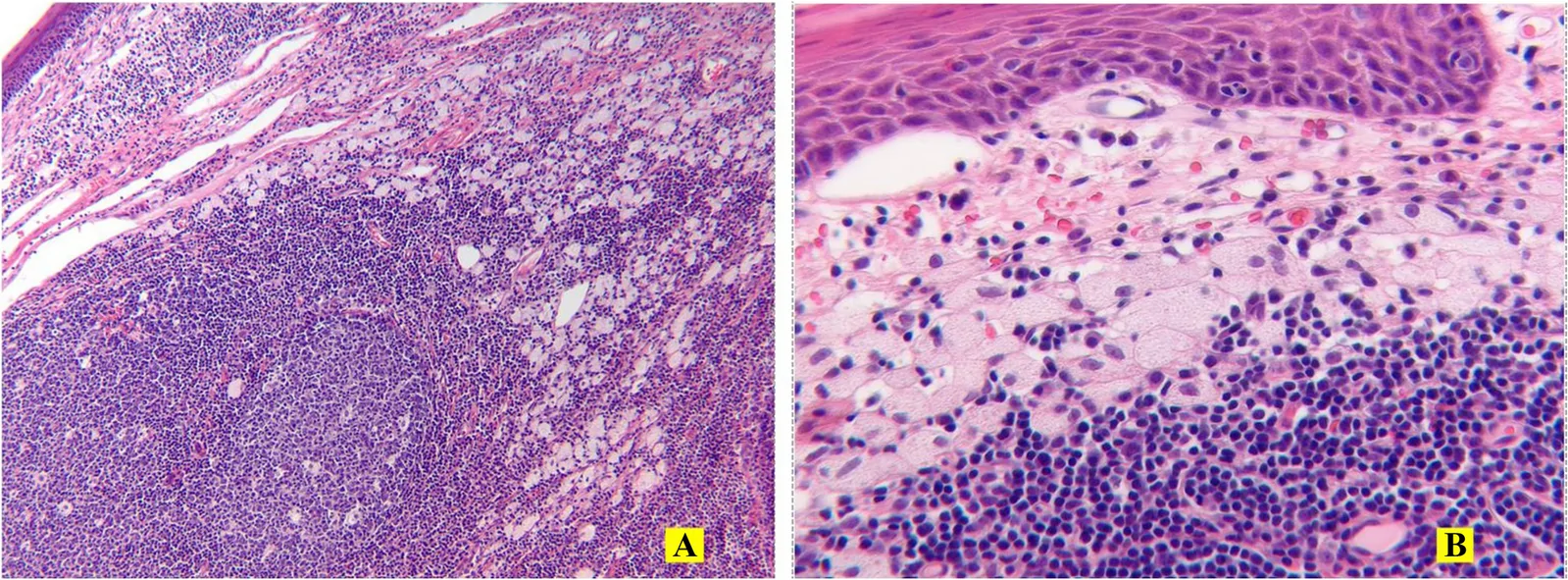
A and B. Histopathology findings of tonsillectomy specimen of Patient 1. (**A**) Low-power magnification showing relative preservation of follicular architecture with follicular hyperplasia of tonsils. (**B**) High-power magnification revealing clusters and sheets of xanthomatous macrophages in the submucosa and infiltrating within the lymphoid parenchyma, a hallmark finding in Tangier disease

A 5-year-old male was referred to the lipid clinic after being diagnosed with TD following adenotonsillectomy performed for obstructive sleep apnea (OSA) secondary to enlarged tonsils and adenoids. Histopathologic examination of the tonsillectomy specimen demonstrated relative preservation of follicular architecture with follicular hyperplasia. Several lymphoid follicles contained active germinal centers with numerous tingible body macrophages. Clusters and sheets of xanthomatous macrophages were present within the submucosa and infiltrated the lymphoid parenchyma. No granulomas or active inflammation were identified (Fig. 1). These unusual findings prompted further evaluation, which revealed markedly reduced HDL-C levels (Table 1).

**Table 1:** Clinical and laboratory parameters of the patients

Patient characteristics	Patient 1	Patient 2
Age at diagnosis (year)	5	11
Presenting symptoms	Enlarged tonsils and adenoids with OSA. Biopsy of tonsillectomy suggested TD.	None; Low HDL-C detected on routine laboratory study
Associated clinical features	Splenomegaly on US abdomen, obesity, mild acanthosis, eczema	Right ventricular conduction delay on ECG and mild MR on echocardiogram due to MASS phenotype.
HDL-C (mg/dL)	< 5 (acceptable level > 45)	3 (acceptable level > 45)
Triglycerides (mg/dL)	84 (acceptable level < 75)	102 (acceptable level < 90)
Total cholesterol (mg/dL)	77 (acceptable level < 170)	85 (acceptable level < 170)
LDL-C (mg/dL)	68 (normal range 50–110)	62 (normal range 50–110)
Genetic work up	Compound heterozygous variant in *ABCA1*: c.1759 C > T, p.Arg587Trp (pathogenic) & c.1733 C > G p.Pro578Arg (VUS)	Homozygous pathogenic variant in *ABCA1-* c.1769G > T p. Trp590Leu

*ECG *Electrocardiogram, *HDL-C-* high density lipoprotein cholesterol, *LDL-C-* low density lipoprotein cholesterol, MASS phenotype- mitral valve prolapse and myopia, an aortic root that may be at the upper limits of normal in caliber, striae atrophicae, and skeletal features reminiscent of Marfan syndrome; OSA- obstructive sleep apnea; TD- Tangier disease; US-ultrasound

The patient was subsequently lost to follow-up and re-presented at 11 years of age with severe obesity, which was attributed to excessive weight gain during the post-COVID-19 period. His medical history was notable for prematurity (35–36 weeks’ gestation) requiring a 13-day neonatal intensive care unit stay for respiratory distress and feeding difficulties, tympanostomy tube placement for recurrent otitis media at 4 years of age, isolated speech delay that improved following tube placement, premature adrenarche beginning at approximately 8.5 years of age, obesity since 9 years of age, and OSA requiring continuous positive airway pressure (CPAP) therapy.

At re-evaluation, physical examination was notable for mild acanthosis nigricans; the remainder of the examination was unremarkable. Neurological examination was normal. Abdominal ultrasonography demonstrated splenomegaly.

Molecular genetic testing using a comprehensive lipid disorder panel was performed because of the characteristic histopathologic findings on the tonsillectomy specimen and markedly reduced HDL-C levels. Genetic testing identified compound *heterozygous* variants in *ABCA1*. One variant, c.1759 C > T (p.Arg587Trp), is a known pathogenic variant previously reported in individuals with TD. The second variant, c.1733 C > G (p.Pro578Arg), is currently not listed in ClinVar and was classified by the testing laboratory as a variant of uncertain significance (VUS). Segregation analysis confirmed that the variants were in trans, consistent with compound heterozygous autosomal recessive inheritance.

Notably, both these variants are located within the first extracellular domain (ECD1) of ABCA1, separated by only nine amino acid residues. This region represents a known mutation-clustering domain in which multiple TD-associated variants (R587W, W590S, Q597R) have been functionally characterized and shown to abolish ApoA-I-mediated HDL assembly [4].

Although the *ABCA1* c.1733 C > G (p.Pro578Arg) variant is currently classified as a VUS, multiple lines of evidence support its pathogenic role in this patient. The variant was identified in trans with a known pathogenic *ABCA1* variant, and the patient demonstrates a highly specific clinical and biochemical phenotype of TD, including extremely low HDL-C levels, characteristic tonsillar histopathology with xanthomatous macrophage infiltration, and splenomegaly. The variant is absent from large population databases, such as gnomAD, and in silico prediction tools (including PolyPhen-2, SIFT, and CADD) consistently predict a deleterious effect on ABCA1 protein function [5–7]. Importantly, the clinical laboratory’s internal data identified at least one additional individual with TD in whom the same c.1733 C > G (p.Pro578Arg) variant was reported in trans with a pathogenic *ABCA1* variant, providing additional clinical evidence supporting its association with disease. Although functional studies are not available for this specific variant, the available clinical, genetic, and computational evidence supports consideration of reclassification of this variant as likely pathogenic under the ACMG/AMP framework [8, 9]. Based on the characteristic clinical, histopathologic, biochemical, and genetic findings, a diagnosis of TD was confirmed in the patient. The patient was referred for multidisciplinary evaluation, including genetics, cardiology, and ophthalmology.

Family testing demonstrated that the patient’s unaffected mother and brother were heterozygous for the pathogenic *ABCA1* variant c.1759 C > T (p.Arg587Trp). Neither had clinical features suggestive of TD, consistent with their carrier status.

We anticipate that inclusion of this case will contribute to future variant reclassification efforts in publicly accessible databases such as ClinVar.

### Patient # 2

A male patient was referred to the lipid clinic at approximately 14 years of age following a recent genetic diagnosis of TD. At approximately 11 years of age, routine laboratory testing performed during a well-child visit by his primary care provider revealed markedly reduced HDL-C levels (Table 1). He was subsequently referred to cardiology and medical genetics. Genetic testing identified a homozygous pathogenic *ABCA1* variant, c.1769G > T (p.Trp590Leu). This variant is classified as pathogenic in ClinVar. It is a missense variant located within the ECD1of the ABCA1 protein, a critical region involved in ApoA-I-mediated HDL assembly. In the homozygous state, this variant has been reported to cause TD.

His past medical history was notable for intermittent abdominal pain and diarrhea, for which he underwent gastroenterology evaluation between 12 and 13 years of age. The evaluation, including upper endoscopy and biopsy, was unremarkable. He denied a history of recurrent throat infections, enlarged tonsils, or tonsillectomy. His family history was noncontributory.

Familial segregation testing was not performed; therefore, the parental genotypes could not be confirmed. Although both parents are presumed to be heterozygous carriers given the homozygous pathogenic ABCA1 variant, alternative mechanisms, including a de novo event or uniparental disomy, cannot be excluded. Neither parent demonstrated clinical or biochemical features suggestive of TD, consistent with the typically asymptomatic phenotype of ABCA1 heterozygotes.

On physical examination, his height was at the 95th percentile, exceeding his mid-parental target height (approximately the 25th percentile). He had arachnodactyly and dolichostenomelia; the remainder of the systemic examination was unremarkable.

Laboratory investigations, including a complete blood count, were normal. Abdominal ultrasonography was normal. Ophthalmologic and neurologic evaluations were also normal. Electrocardiography demonstrated a benign right ventricular conduction delay. Echocardiography performed at approximately 14 years of age showed mild buckling of the anterior mitral valve leaflet with mild mitral regurgitation. These findings remained stable on repeat echocardiography at 15 years of age. Given the mitral valve abnormalities and skeletal features, a diagnosis of MASS phenotype (mitral valve prolapse, myopia, an aortic root at the upper limits of normal, striae atrophicae, and skeletal features resembling Marfan syndrome) was considered [10]. Coronary computed tomography angiography (CCTA) and an exercise stress test were normal, and the mitral valve findings were attributed to the MASS phenotype. To our knowledge, there is no published evidence supporting an association between TDand the MASS phenotype or other hereditary connective tissue disorders. Therefore, these findings were considered an incidental coexisting condition rather than a recognized manifestation of TD.

He continues multidisciplinary follow-up with endocrinology, gastroenterology, ophthalmology, cardiology, and neurology and remained asymptomatic at his most recent follow-up at 15 years of age.

## Pathophysiology

**Fig. 2:**
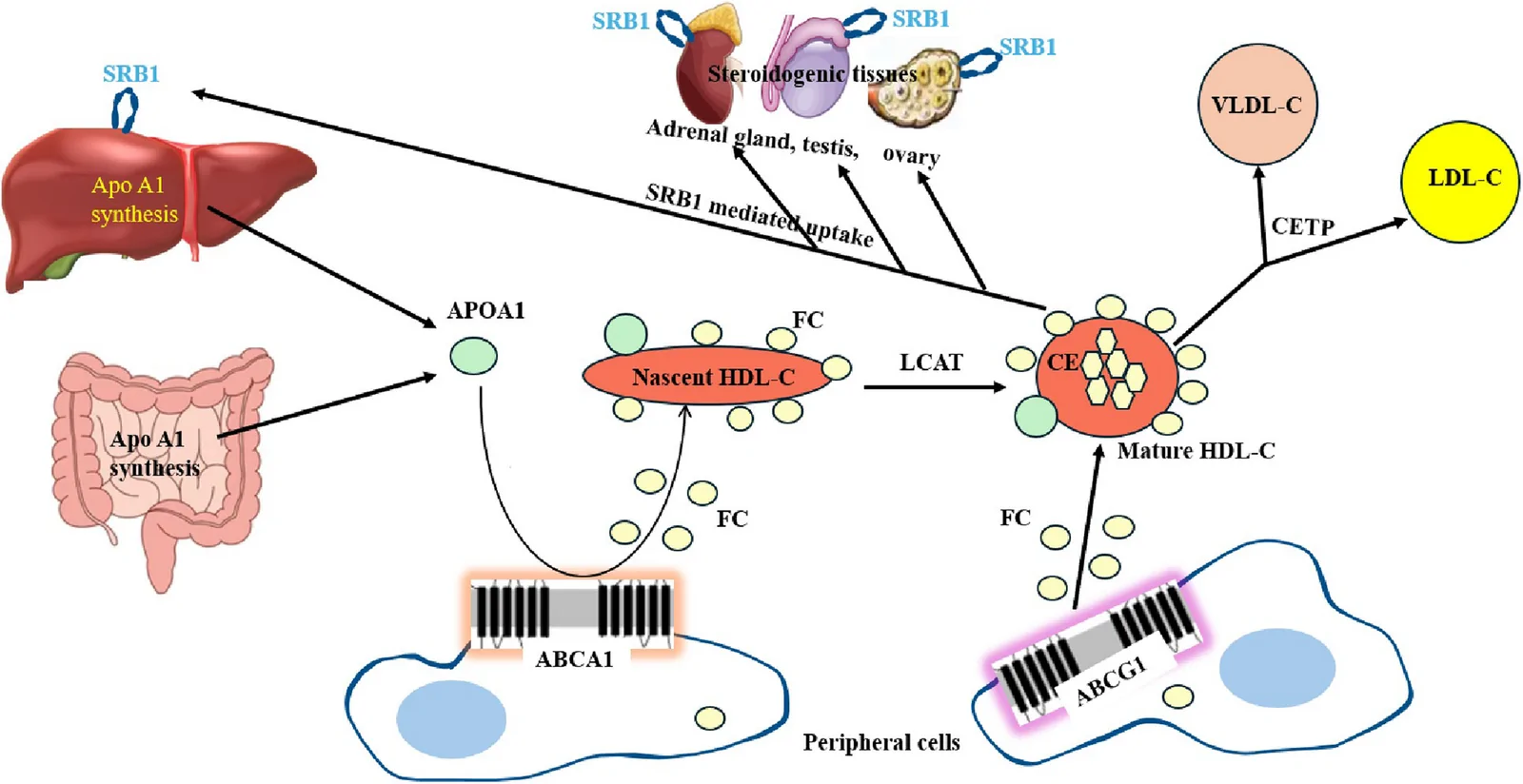
Pathophysiology of impaired reverse cholesterol transport in Tangier disease. In normal physiology, lipid-poor apolipoprotein A-I (ApoA1), synthesized primarily in the liver and intestine, accepts cellular cholesterol (free cholesterol, FC) and phospholipids from peripheral cells and macrophages through the ATP-binding cassette transporter A1 (ABCA1), forming nascent high-density lipoprotein (HDL-C). Lecithin–cholesterol acyltransferase (LCAT) esterifies free cholesterol (cholesteryl ester, CE), allowing maturation of HDL-C particles. ABCG1 facilitates cholesterol efflux from cells to mature HDL-C. Mature HDL-C facilitates reverse cholesterol transport by delivering cholesterol to the liver and steroidogenic tissues (adrenal glands, testes, and ovaries) via scavenger receptor class B type 1 (SR-B1). Cholesterol may also be indirectly returned to the liver through exchange with apoB-containing lipoproteins (VLDL and LDL) mediated by cholesterol ester transfer protein (CETP) In Tangier disease, loss-of-function variants in ABCA1 impair cholesterol efflux to ApoA1, resulting in rapid ApoA1 catabolism, markedly reduced HDL-C levels, and defective reverse cholesterol transport. This leads to intracellular cholesterol accumulation in reticuloendothelial and peripheral tissues, contributing to characteristic clinical features including xanthomatous macrophage infiltration, hepatosplenomegaly, peripheral neuropathy, corneal opacities, and increased risk of premature atherosclerotic cardiovascular disease

HDL-C plays a key role in reverse cholesterol transport (RCT) (Fig. 2). It transfers cholesterol from peripheral tissues as well as atherosclerotic plaques to liver and organs of steroidogenesis (gonads and adrenal cortex) via scavenger receptor B1(SRB1). In the liver cholesterol is metabolized into bile acids for excretion via bile. HDL-C also exchanges cholesterol for triglycerides with apo B particles such as low-density lipoprotein cholesterol (LDL-C) facilitated by cholesterol ester transfer protein (CETP) thereby indirectly transferring cholesterol back to liver via LDL receptor [11].

ApoA-I, the main structural protein of HDL is synthesized predominantly in liver and intestine. Cellular cholesterol and phospholipid efflux to the lipid poor ApoA-I (pre- beta HDL) mediated by ABCA1 protein, forms nascent HDL-C. ABCA1 is a 12 transmembrane ATP binding cassette transporter protein encoded by *ABCA1*. Lecithin cholesterol acyl transferase (LCAT) esterifies free cholesterol on the surface of nascent HDL to cholesterol ester which then moves to the center of HDL forming mature HDL. ABCG1 facilitates cholesterol efflux from cells to mature HDL-C [11, 12]. Loss of function variants in *ABCA1* leads to poor lipidation of newly formed ApoA-I resulting in its rapid catabolism (12 h in TD vs. more than 60 h in healthy controls) and hence very low HDL-C [1]. The same pathophysiologic mechanism also contributes to reduced ApoA-II concentrations in TD through accelerated clearance of poorly lipidated ApoA-II from the circulation by renal filtration and the reticuloendothelial system [13, 14].

Very low HDL-C affects RCT resulting in intracellular cholesterol accumulation in various tissues especially reticuloendothelial cells producing lipid-laden “foam cells” — a process termed xanthomatous macrophage infiltration. This pattern of lipid deposition is fundamentally distinct from the classic cutaneous, tendon, or eruptive xanthomas observed in disorders of LDL metabolism such as familial hypercholesterolemia, and true cutaneous xanthomas are not a characteristic clinical feature of TD [15]. The xanthomatous macrophage infiltration is responsible for the characteristic clinical presentation noted in TD such as hyperplastic yellow-orange tonsils, hepatomegaly and splenomegaly [1, 8, 11]. Neuropathy in TD results from the accumulation of cholesteryl esters within Schwann cells and other nerve cells [16]. Similarly, cholesterol accumulation in cornea produces corneal opacities. Impaired RCT may be responsible for the increased risk for ASCVD seen in patients with TD, even when the LDL-C is low [17].

Altered lipid rafts may also contribute to the development of premature ASCVD. Lipid rafts are cholesterol rich microdomains in the plasma membrane that regulate intracellular and extracellular signals. ABCA1 deficiency causes increase in lipid rafts which in turn is believed to cause abnormal cytokine release. Prior studies demonstrated abnormal LDL-C in patients with TD; TG rich, cholesterol and phospholipid poor LDL-C like particles that were smaller in size compared to normal LDL-C. Gradual accrual of these abnormal lipoproteins overtime may contribute to the development of ASCVD in these patients [17]. Additionally, decreased insulin secretion from pancreatic beta cells has been reported in heterozygous carriers of TD. All these metabolic derangements may contribute to increased ASCVD risk [3, 16].

Hypertriglyceridemia observed in TD may be related to reduced lipoprotein lipase (LPL) activity secondary to elevated angiopoietin-related protein 3 (ANGPTL3), poor adipose tissue storage capacity secondary to cholesterol accumulation in adipose tissue, and enhanced VLDL secretion in the context of hepatic steatosis [1, 18]. In ABCA1 deficiency, impaired cholesterol efflux from hepatocytes leads to intracellular cholesterol accumulation, which may activate liver X receptor (LXR)-mediated transcription of ANGPTL3 [19, 20]. Elevated ANGPTL3 inhibits LPL and endothelial lipase (EL), impairing the clearance of triglyceride-rich lipoproteins [21]. In TD, the near-complete absence of HDL may alter the distribution and activity of ANGPTL3, potentially contributing to dysregulated triglyceride metabolism [22].

Additionally, decreased hepatic lipase (HL) activity that facilitates the hydrolysis of triglycerides and phospholipids in intermediate density lipoprotein (IDL), LDL-C and HDL-C is noted in patients with TD. HL is synthesized in liver and remains bound to heparan sulfate proteoglycans (HSPG) on hepatocytes and hepatic endothelial cells in the inactive form. HL release from HSPG is mediated by HDL-C along with its apolipoproteins ApoA-II and ApoE. Post prandially ApoA-II stimulates the release of HL and during fasting ApoE rich HDL-C inhibits its release into circulation. Because TD is characterized by a severe deficiency in both HDL-C and ApoA-II, the necessary triggers for HL release are absent, leading to the low HL activity observed in these patients [23].

Lipid accumulation in macrophages along with reduced cholesterol efflux can cause low red blood cell (RBC) membrane cholesterol/phospholipid ratio and increased phosphatidylcholine leading to abnormally shaped RBCs (poikilocytes) especially stomatocytes with reduced surface/volume ratio in TD. Premature destruction of these fragile RBCs causes hemolytic anemia [24, 25]. ABCA1 deficiency can cause impaired platelet production, release and responsiveness to definite stimuli resulting in thrombocytopenia. Furthermore, associated splenomegaly may also cause thrombocytopenia and anemia in these patients [26].

## Clinical Features

Clinical manifestations of TD may vary based on cholesterol accumulation in different tissues [11, 26]. Onset can occur any time between 2 and 67 years of age. Cardinal clinical features include enlarged orange-yellow tonsils, hepatosplenomegaly, lymphadenopathy, corneal opacities, ASCVD, neuropathy, anemia and thrombocytopenia [1, 27]. Enlarged tonsils and corneal opacity often manifest in the first decade of life and a history of recurrent tonsillitis or tonsillectomy may be present oftentimes [3].

Neuropathic symptoms may be observed in about 50% of patients [27]. Neuropathy can manifest as syringomyelia-like neuropathy, resulting in a slow, progressive weakness, muscle wasting. It typically results in loss of pain and temperature sensation affecting distal upper limb muscles, typically sparing the lower extremities. Some patients experience a multifocal mono- or polyneuropathy, characterized by relapsing-remitting pattern of motor and sensory loss. While some cases may be subclinical, others present as distal symmetric polyneuropathies or manifest as chronic neuropathic pain [28].

Premature ASCVD is another serious manifestation of TD which eventuates in the sixth or seventh decades of life and is often seen in patients with LDL-C levels exceeding 70 mg/dL [29].

Rare presentations include primary ovarian insufficiency and infertility, bone marrow involvement with diffuse histiocytosis, mitral valvulopathy resulting from cholesterol accumulation, gastrointestinal manifestations such as chronic diarrhea or gastrointestinal bleeding, hearing loss and skin lesions such as prurigo nodularis, skin ulcer, and painless scald or burn scars [1, 11, 26, 30, 31].

Heterozygous carriers of TD are usually asymptomatic but have biochemical abnormalities with HDL-C levels around 50th percentile of normal and normal or slightly elevated triglycerides [27]. Carriers of TD are at increased risk of ASCVD given their low HDL-C levels and hence requires regular follow up [8, 32].

The long-term consequences of TD are variable and heterogeneous, driven by progressive cholesterol ester accumulation in macrophages and the reticuloendothelial system due to dysfunctional ABCA1-mediated cholesterol efflux. TD is generally associated with good prognosis which relies chiefly on development and advancement of neuropathy and/or ASCVD [33].

## Differential Diagnosis

Hypoalphalipoproteinemia can be primary (genetic) or secondary (acquired). Acquired causes of hypoalphalipoproteinemia include hypertriglyceridemia, severe sepsis, inflammatory conditions such as systemic lupus erythematosus and rheumatoid arthritis, monoclonal gammopathies, advanced liver disease, medications such as beta-blockers, benzodiazepines and exogenous testosterone treatment. Primary or genetic cause of hypoalphalipoproteinemia other than TD include familial ApoA-I deficiency and familial LCAT deficiency(Table 2) [34–37]. LCAT deficiency is characterized by progressive corneal opacification which is more severe than that seen in TD and ApoA-I deficiency, anemia secondary to increased red cell fragility resulting from abnormal cell membrane lipid composition and early and progressive chronic kidney disease. While cutaneous xanthoma are commonly seen in ApoA-I deficiency, enlarged orange yellow tonsils and peripheral neuropathy are distinct features of TD [3, 36, 38].

**Table 2:** Familial causes of hypoalphalipoproteinemia

	Tangier disease	Familial APOA1 deficiency	Familial LCAT deficiency
Mode of inheritance	AR	AR	AR
HDL-C	< 5 mg/dL usually and rarely 5–10 mg/dL	< 5 mg/dL usually	< 10 mg/dL
ApoA-I	< 30 mg/dL usually and typically < 5 mg/dL	Undetectable	< 40 mg/dL
LCAT level	Normal	Normal, but decreased activity	Undetectable
Triglycerides	Mild increase	Normal or decreased	Moderate increase
LDL-C	50% of the normal	Normal	50% of the normal
CE)/TC	No relative increase in FC, normal CE/TC (about 70%)	Relative increase in FC, low CE/TC	Relative increase in FC, low CE/TC (< 25%)
Characteristic clinical features	**Orange tonsils**, Hepatosplenomegaly Corneal opacity (mild) **Neuropathy** CVD	Corneal opacity (mild to moderate) **Xanthoma** CVD	Corneal opacity (marked), anemia, **proteinuria**,** progressive renal disease**
Molecular genetic testing	Homozygous/compound heterozygous pathogenic variant in *ABCA1*	Homozygous/compound heterozygous pathogenic variant in *ApoA1*	Homozygous/compound heterozygous pathogenic variant in *LCAT*

*ABCA1* adenosine triphosphate binding cassette subfamily A member 1, *ApoA-I* apolipoprotein A1, *AR* autosomal recessive, *CVD* cardiovascular disease, *CKD* chronic kidney disease, *CE *cholesteryl ester, *FC *free cholesterol, *HDL-C* high density lipoprotein cholesterol, *LCAT *lecithin cholesterol acyl transferase deficiency, *TC *total cholesterol

## Diagnosis

TD is diagnosed based on characteristic clinical features, laboratory tests, and molecular genetic testing for *ABCA1*. Characteristic lipid panels of these patients include HDL-C < 5 mg/dL usually and rarely 5–10 mg/dL, ApoA-I concentration < 30 mg/dL usually and typically < 5 mg/dL, total cholesterol < 150 mg/dL, hypertriglyceridemia up to 400 mg/dL, and LDL-C that is 40–50% lower than normal [26].

Current pediatric guidelines recommend universal lipid screening once between 9 and 11 years of age and again between 17 and 21 years of age, with earlier targeted screening for children with cardiovascular risk factors or a family history of dyslipidemia. Although TD is rare, universal lipid screening may facilitate its incidental detection through recognition of profoundly reduced HDL-C concentrations, particularly in otherwise asymptomatic individuals. This is illustrated by our second patient, whose diagnosis was prompted by the incidental finding of markedly reduced HDL-C during routine lipid screening. Recognition of extremely low HDL-C levels should prompt consideration of inherited disorders of HDL metabolism, including TD, and appropriate genetic evaluation [39].

## Management

Currently there is no definite treatment for TD. It’s managed with measures aimed at preventing cholesterol accumulation in various tissues there by slowing disease progression. These measures include a healthy heart diet, regular physical activity, smoking cessation, control of blood pressure and diabetes mellitus and lipid lowering medications such as statins [27]. Statin therapy in TD is recommended for its LDL-C lowering effect, its pleiotropic anti-inflammatory properties that attenuate the heightened vascular inflammation inherent to ABCA1 deficiency, and its role in comprehensive ASCVD risk factor management in a population with a moderately elevated cardiovascular risk [15]. Although therapies that increase HDL-C levels, including niacin, fibrates, and cholesteryl ester transfer protein (CETP) inhibitors, have been investigated, increases in HDL-C have not consistently translated into reductions in cardiovascular events in clinical trials [38, 40]. The role of these therapies specifically in TD remains uncertain, and there are limited data to establish their efficacy in reducing cardiovascular events in this rare disorder.

Present treatment modalities focus on symptom management: tonsillectomy in cases of airway obstruction, obstructive sleep apnea or pressure symptoms such as dysphagia, corneal transplantation for corneal opacities that affect day to day activities and management of hemolytic anemia and thrombocytopenia [33]. There have been isolated reports of improvement in neuropathy with steroids, azathioprine, intravenous immunoglobulin therapy and glycosphingolipid synthesis inhibitor, miglustat [27, 41]. Patients with neuropathy may require adjuvant physiotherapy and ankle foot orthoses. Neurotoxic medications should be avoided in these patients. Patients with hepatosplenomegaly should be advised to avoid contact sports to prevent the risk of splenic rupture [33].

Genetic counseling should be provided to the patients and their families who are carriers or are at risk of being carriers. Cascade testing or targeted testing for the same genetic mutation among family members of the proband should be offered permitting early diagnosis, prevention, or management before symptoms appear, decreasing morbidity and mortality. Additionally, prenatal as well as preimplantation genetic testing should be offered [33].

## Surveillance

There is no pediatric centric recommendations for surveillance of patients with TD diagnosed during childhood and current recommendations focus on adult patients. These patients require annual neurology evaluation with nerve conduction studies and electromyogram (EMG) as needed [27]. Annual ophthalmological evaluation is needed given the risk of age-related corneal opacities in patients with TD [28]. Patients should be evaluated for hepatosplenomegaly by physical examination at each visit and abdominal ultrasound may be considered as needed. They should be monitored for hematologic manifestations such as anemia and thrombocytopenia with complete blood counts and peripheral smear during routine check-ups [27, 33]. Patients with TD are at increased risk of developing early ASCVD usually after age 40 years with risk increasing with advancing age. Current data recommend annual cardiology follow-up with echocardiogram. Coronary computed tomography angiography (CCTA) and carotid duplex ultrasonography should be obtained as needed TD [27].

## Limitations

This report has several limitations. Although multiple lines of evidence support the pathogenicity of the ABCA1 c.1733 C > G (p.Pro578Arg) variant in Patient 1 — including its identification in trans with a known pathogenic variant, absence from population databases, deleterious in silico predictions, and a highly characteristic clinical, biochemical, and histopathologic phenotype — formal functional studies, such as cholesterol efflux assays, were not performed. We therefore cannot definitively exclude the possibility that this represents a private familial variant rather than a disease-causing one, and reclassification will ultimately require functional validation and additional reported cases. In Patient 2, familial genetic testing was not performed, although both parents are presumed obligate heterozygous carriers given the homozygous state of the pathogenic variant. Finally, this is a narrative review based on two patients and may not capture the full phenotypic spectrum of TD.

## Conclusions

TD is an extremely uncommon disease with wide heterogeneity in clinical presentation and progression, characterized by marked deficiency or absence of HDL-C and ApoA-I resulting from homozygous or compound heterozygous pathogenic variant in *ABCA1*. Very low HDL-C affects RCT resulting in intracellular cholesterol accumulation in various tissues especially reticuloendothelial cells producing characteristic clinical presentation noted in TD. Currently there is no definite treatment for TD.

## Key References

Important:

- Koseki M, Yamashita S, Ogura M, Ishigaki Y, Ono K, Tsukamoto K, et al. Current diagnosis and management of tangier disease. J Atheroscler Thromb. 2021;28(8):802–10.
  - A review article discussing the clinical features, pathophysiology, approach to diagnosis and management of TD.
- Moussa S, Price J, Frye J, Chen O, Rahman T. Primary Hypoalphalipoproteinemia With Significant Premature Atherosclerosis. JACC Case Rep. 2024;29(23):102716.
  - A case report describing an otherwise healthy and asymptomatic young woman with significant coronary artery disease secondary to TD which was managed with statin.

Very important:

- Semmler G, Baumgartner C, Metz M, Gensluckner S, Habisch H, Hofer H, et al. Lipid Dysregulation in Tangier Disease: A Case Series and Metabolic Characterization. J Clin Endocrinol Metab. 2025;110 [7]:e2146–56.
  - A multicenter cohort study investigating the phenotype and lipid metabolism in TD.
- Sphitzen S, Golomb M, Mowaswes M, Bitzur R, Horowitz Cederboim S, Leker RR, et al. Missing in action: the genetic mysteries of extremely low HDL cholesterol. Front Cardiovasc Med. 2025;12:1553259.
  - This study discusses three patients with pathogenic mutations associated with genetic low HDL-C syndrome, including *ABCA1*, *LCAT and APOA1* and provides a detailed analysis of the clinical and biochemical profiles and disease progression of these patients.
- Furuta Y, Osaki Y, Nakagawa Y, Han SI, Araki M, Shikama A, et al. Genetic and Functional Analyses of Patients with Marked Hypo-High-Density Lipoprotein Cholesterolemia. J Atheroscler Thromb. 2024;31 [9]:1304–18.
  - Analyzes 2 patients with TD and discusses the molecular mechanism causing hypo HDL cholesterolemia in these patients.
- Öztürk S, Doǧan ME, Kadloǧlu Yllmaz B, Güleç A, Soylu Üstkoyuncu P, Kardaş F, et al. The clinical presentation and genetic diagnosis of Tangier disease in the pediatric age group. J Pediatr Endocrinol Metab. 2025;38 [3]:271–8.
  - A retrospective and cross sectional study describing clinical characteristics of patients diagnosed with TD in childhood and thereby aiming to enhance early diagnosis of asymptomatic cases.

## Data Availability

No datasets were generated or analysed during the current study.

## References

[1] Semmler G, Baumgartner C, Metz M, Gensluckner S, Habisch H, Hofer H, et al. Lipid dysregulation in tangier disease: a case series and metabolic characterization. J Clin Endocrinol Metab. 2025;110(7):e2146–56. 40037526 10.1210/clinem/dgaf131 PMC12187588

[2] Fredrickson DS, Young O, Shiratori T, Briggs N. The inheritance of high density lipoprotein deficiency (Tangier Disease) *. J Clin Invest. 1964;43(2):228–36. 14162531 10.1172/JCI104907 PMC289516

[3] Koseki M, Yamashita S, Ogura M, Ishigaki Y, Ono K, Tsukamoto K, et al. Current diagnosis and management of tangier disease. J Atheroscler Thromb. 2021;28(8):802–10. 33994407 10.5551/jat.RV17053 PMC8326168

[4] Tanaka AR, Abe-Dohmae S, Ohnishi T, Aoki R, Morinaga G, Okuhira K, ichiro, et al. Effects of mutations of ABCA1 in the first extracellular domain on subcellular trafficking and ATP binding/hydrolysis. J Biol Chem. 2003;278(10):8815–9. 12509412 10.1074/jbc.M206885200

[5] Bertolini S, Pisciotta L, Seri M, Cusano R, Cantafora A, Calabresi L, et al. A point mutation in ABC1 gene in a patient with severe premature coronary heart disease and mild clinical phenotype of Tangier disease. Atherosclerosis [Internet]. 2001;154(3):599–605. Available from:. www.elsevier.com/locate/atherosclerosis. 11257260 10.1016/s0021-9150(00)00587-6

[6] Pisciotta L, Bocchi L, Candini C, Sallo R, Zanotti I, Fasano T, et al. Severe HDL deficiency due to novel defects in the ABCA1 transporter. J Intern Med. 2009;265(3):359–72. 19019193 10.1111/j.1365-2796.2008.02019.x

[7] Théaudin M, Couvert P, Fournier E, Bouige D, Bruckert E, Perrotte P, et al. Lewis-sumner syndrome and tangier disease. Arch Neurol. 2008;65(7):968–70. 18625867 10.1001/archneur.65.7.968

[8] Teigen M, Ølnes ÅS, Bjune K, Bogsrud MP, Strøm TB. Functional characterization of genetic variants affecting the intracellular domains of ATP-binding cassette transporter A1 (ABCA1). J Lipid Res. 2025;66(8):100854. 40617357 10.1016/j.jlr.2025.100854 PMC12341595

[9] Richards S, Aziz N, Bale S, Bick D, Das S, Gastier-Foster J, et al. Standards and guidelines for the interpretation of sequence variants: a joint consensus recommendation of the American College of Medical Genetics and Genomics and the Association for Molecular Pathology. Genet Med. 2015;17(5):405–24. 10.1038/gim.2015.30 PMC4544753 25741868

[10] Pyeritz RE. Evaluation of the adolescent or adult with some features of Marfan syndrome. Genet Med. 2012;14(1):171–7. 22237449 10.1038/gim.2011.48

[11] Sphitzen S, Golomb M, Mowaswes M, Bitzur R, Horowitz Cederboim S, Leker RR, et al. Missing in action: the genetic mysteries of extremely low HDL cholesterol. Front Cardiovasc Med. 2025;12:1553259. 40476138 10.3389/fcvm.2025.1553259 PMC12137234

[12] Furuta Y, Osaki Y, Nakagawa Y, Han SI, Araki M, Shikama A, et al. Genetic and functional analyses of patients with marked hypo-high-density lipoprotein cholesterolemia. J Atheroscler Thromb. 2024;31(9):1304–18. 38538338 10.5551/jat.64579 PMC11374561

[13] Kay LL, Ronan R, Schaefer EJ, Bryan Brewer H. Tangier disease: A structural defect in apolipoprotein A-I (apoA-ITangir) (apolipoprotein A 1H/dyslipoproteinemia/atherosclerosis/high density lipoproteins/cholesterol). Proc Natl Acad Sci U S A. 1982;79(8):2485–9. 6806810 10.1073/pnas.79.8.2485 PMC346223

[14] Schaefer EJ, Blum CB, Levy RI, Jenkins LL, Alaupovic P, Foster DM, et al. Metabolism of high-density lipoprotein apolipoproteins in Tangier disease. N Engl J Med. 1978;299(17):905–10. 211412 10.1056/NEJM197810262991701

[15] Hegele RA, Borén J, Ginsberg HN, Arca M, Averna M, Binder CJ, et al. Rare dyslipidaemias, from phenotype to genotype to management: a European Atherosclerosis Society task force consensus statement. Lancet Diabetes Endocrinol. 2020;8(1):50–67. 31582260 10.1016/S2213-8587(19)30264-5

[16] Ogura M. HDL, cholesterol efflux, and ABCA1: Free from good and evil dualism. J Pharmacol Sci. 2022;150(2):81–9. 36055755 10.1016/j.jphs.2022.07.004

[17] Murano T, Yamaguchi T, Tatsuno I, Suzuki M, Noike H, Takanami T, et al. Subfraction analysis of circulating lipoproteins in a patient with Tangier disease due to a novel ABCA1 mutation. Clin Chim Acta. 2016;452:167–72. 26616730 10.1016/j.cca.2015.11.021

[18] Bi X, Pashos EE, Cuchel M, Lyssenko NN, Hernandez M, Picataggi A, et al. ATP-binding cassette transporter A1 deficiency in human induced pluripotent stem cell-derived hepatocytes abrogates HDL biogenesis and enhances triglyceride secretion. EBioMedicine. 2017;18:139–45. 28330813 10.1016/j.ebiom.2017.03.018 PMC5405159

[19] Kaplan R, Zhang T, Hernandez M, Xiaodong Gan F, Wright SD, Waters MG, et al. Regulation of the angiopoietin-like protein 3 gene by LXR. J Lipid Res. 2003;44(1):136–43. 12518032 10.1194/jlr.m200367-jlr200

[20] Inaba T, Matsuda M, Shimamura M, Takei N, Terasaka N, Ando Y, et al. Angiopoietin-like protein 3 mediates hypertriglyceridemia induced by the liver X receptor. J Biol Chem. 2003;278(24):21344–51. 12672813 10.1074/jbc.M213202200

[21] Kersten S. Angiopoietin-like 3 in lipoprotein metabolism. Nat Rev Endocrinol. 2017;13(12):731–9. 28984319 10.1038/nrendo.2017.119

[22] Kraaijenhof JM, Tromp TR, Nurmohamed NS, Reeskamp LF, Langenkamp M, Levels JHM, et al. ANGPTL3 (Angiopoietin-Like 3) preferentially resides on high-density lipoprotein in the human circulation, affecting its activity. Am Heart Assoc. 2023;12(21):e030476. 10.1161/JAHA.123.030476 PMC10727379 37889183

[23] Chatterjee C, Sparks DL. Hepatic lipase, high density lipoproteins, and hypertriglyceridemia. Am J Pathol. 2011;178(4):1429–33. 21406176 10.1016/j.ajpath.2010.12.050 PMC3078429

[24] Chiriac R. Expect the unexpected: Tangier disease and monoclonal gammopathy of undetermined significance. Br J Haematol. 2025;206(6):1542–3. 40302156 10.1111/bjh.20096

[25] Reinhart WH, Gössi U, Bütikofer P, Ott P, Sigrist H, Schatzmann H-J, et al. Haemolytic anaemia in analpha‐lipoproteinaemia (Tangier disease): morphological, biochemical, and biophysical properties of the red blood cell. Br J Haematol. 1989;72(2):272–7. 2757970 10.1111/j.1365-2141.1989.tb07694.x

[26] Babayeva A, Cerit ET, Kayhan G, Inan A, Akturk M. Is Tangier disease a rare cause of premature ovarian insufficiency?: a case report. J Clin Lipidol. 2025;19(4):1158–63. 40360373 10.1016/j.jacl.2025.03.011

[27] Öztürk S, Doǧan ME, Kadloǧlu Yllmaz B, Güleç A, Soylu Üstkoyuncu P, Kardaş F, et al. The clinical presentation and genetic diagnosis of Tangier disease in the pediatric age group. J Pediatr Endocrinol Metab. 2025;38(3):271–8. 39817629 10.1515/jpem-2024-0335

[28] Ruiz-Ospina E, Ortiz-Corredor F, Milena Castellar-Leones S, Delgado-Martinez JR. Quantitative sensory testing in a girl with tangier disease: a case report. Cureus. 2025;17(4):e81676. 40330358 10.7759/cureus.81676 PMC12050346

[29] Malick WA, Schaefer EJ, Hegele RA, Rosenson RS. ABCA1 deficiency: a rare cause of premature coronary artery disease. JACC Case Rep. 2023;18:101904. 37545679 10.1016/j.jaccas.2023.101904 PMC10401051

[30] Ramalho AR, Moreira S, Ramos LC, de Moura JP. A novel variant in the ABCA1 gene for Tangier disease with diffuse histiocytosis of bone marrow. J Clin Lipidol. 2025;19(2):372–6. 39863479 10.1016/j.jacl.2024.12.008

[31] Mercan M, Yayla V, Altinay S, Seyhan S. Peripheral neuropathy in Tangier disease: A literature review and assessment. J Peripher Nerv Syst. 2018;23(2):88–98. 29582519 10.1111/jns.12265

[32] Patel SB, Belalcazar LM, Afreen S, Balderas R, Hegele RA, Karpe F, et al. American association of clinical endocrinology consensus statement: algorithm for management of adults with dyslipidemia – 2025 update. Endocr Pract. 2025;31(10):1207–38. 40938233 10.1016/j.eprac.2025.07.014

[33] Nlm Citation:, Burnett JR, Hooper AJ, Mccormick SP. Tangier Disease. GeneReviews^®^ [Internet] Seattle (WA). University of Washington, Seattle. 2019:1993–2025. 31751110

[34] Moussa S, Price J, Frye J, Chen O, Rahman T. Primary hypoalphalipoproteinemia with significant premature atherosclerosis. JACC Case Rep. 2024;29(23):102716. 39691320 10.1016/j.jaccas.2024.102716 PMC11646917

[35] Shamburek RD, Bakker-Arkema R, Auerbach BJ, Krause BR, Homan R, Amar MJ, et al. Familial lecithin:cholesterol acyltransferase deficiency: first-in-human treatment with enzyme replacement. J Clin Lipidol. 2016;10(2):356–67. 27055967 10.1016/j.jacl.2015.12.007 PMC4826469

[36] Santos RD, Asztalos BF, Martinez LRC, Miname MH, Polisecki E, Schaefer EJ. Clinical presentation, laboratory values, and coronary heart disease risk in marked high-density lipoprotein-deficiency states. J Clin Lipidol. 2008;2(4):237–47. 21291740 10.1016/j.jacl.2008.06.002 PMC6942923

[37] Santos RD, Schaefer EJ, Asztalos BF, Polisecki E, Wang J, Hegele RA, et al. Characterization of high density lipoprotein particles in familial apolipoprotein A-I deficiency. J Lipid Res. 2008;49(2):349–57. 17991756 10.1194/jlr.M700362-JLR200

[38] Bonilha I, Luchiari B, Nadruz W, Sposito AC. Very low HDL levels: clinical assessment and management. Arch Endocrinol Metab. 2023;67(1):3–18. 36651718 10.20945/2359-3997000000585 PMC9983789

[39] Peterson AL, Ashraf AP, Bachman J, Hegele RA, Rashad M, South AM, et al. Screening, diagnosis, and management of pediatric hypertriglyceridemia: a scientific statement from the American Heart Association. Arterioscler Thromb Vasc Biol. 2026;46(4):e000195. 41705332 10.1161/ATV.0000000000000195

[40] Prineas RJ, Friedewald W. Fifteen year mortality in coronary drug project patients: long-term benefit with niacin. J Am Coll Cardiol. 1986;8(6):1245–55. 3782631 10.1016/s0735-1097(86)80293-5

[41] Karabudak S, Güzel V, Güler B, Uyanık B, Gürsoy AE. A case report of Tangier disease presents with acute sensorimotor polyneuropathy and its treatment approach. J Clin Lipidol. 2024;18(2):e285–9. 38172008 10.1016/j.jacl.2023.11.015

